# Impacts of Diet and Exercise on Maternal Gut Microbiota Are Transferred to Offspring

**DOI:** 10.3389/fendo.2018.00716

**Published:** 2018-11-29

**Authors:** Shyam Prakaash Bhagavata Srinivasan, Mukesh Raipuria, Hasnah Bahari, Nadeem O. Kaakoush, Margaret J. Morris

**Affiliations:** School of Medical Sciences, Faculty of Medicine, UNSW Sydney, Sydney, NSW, Australia

**Keywords:** maternal obesity, voluntary exercise, programming, gut microbiome, adiposity, birthweight

## Abstract

**Background:** It is well established that maternal exercise during pregnancy improves metabolic outcomes associated with obesity in mothers and offspring, however, its effects on the gut microbiota of both mother and offspring, are unknown. Here, we investigated whether wheel running exercise prior to and during pregnancy and prolonged feeding of an obesogenic diet were associated with changes in the gut microbiomes of Sprague-Dawley rat dams and their offspring. Female rats were fed either chow or obesogenic diet, and half of each diet group were given access to a running wheel 10 days before mating until delivery, while others remained sedentary. 16S rRNA gene amplicon sequencing was used to assess gut microbial communities in dams and their male and female offspring around the time of weaning.

**Results:** Statistical analyses at the operational taxonomic unit (OTU) level revealed that maternal obesogenic diet decreased gut microbial alpha diversity and altered abundances of bacterial taxa previously associated with obesity such as *Bacteroides* and *Blautia* in dams, and their offspring of both sexes. Distance based linear modeling revealed that the relative abundances of *Bacteroides* OTUs were associated with adiposity measures in both dams and offspring. We identified no marked effects of maternal exercise on the gut microbiota of obesogenic diet dams or their offspring. In contrast, maternal exercise decreased gut microbial alpha diversity and altered the abundance of 88 microbial taxa in offspring of control dams. Thirty of these taxa were altered in a similar direction in offspring of sedentary obesogenic vs. control diet dams. In particular, the relative abundances of *Oscillibacter* OTUs were decreased in offspring of both exercised control dams and sedentary obesogenic diet dams, and associated with blood glucose concentrations and adiposity measures. Analyses of predicted bacterial metabolic pathways inferred decreased indole alkaloid biosynthesis in offspring of both obesogenic diet and exercised control dams.

**Conclusions:** Our data suggest that maternal exercise prior to and during pregnancy resulted in gut dysbiosis in offspring of control dams. Importantly, alterations in the maternal gut microbiota by obesogenic diet or obesity were transferred to their offspring.

## Introduction

The worldwide incidence of overweight and obesity among children and adults has nearly tripled since 1975 (1). Overweight and obesity are usually described using the Body Mass Index (BMI); BMI scores ≥25 and ≥30 kg/m^2^ are classified as overweight and obese, respectively. The strong increase in obesity prevalence has affected as many as 50% of women of reproductive age in Western countries (2, 3). In addition to increasing the risk of several complications during pregnancy (4), maternal obesity can have long term detrimental consequences on offspring, including greater likelihood to develop childhood and adult obesity (5, 6). Physical exercise during pregnancy may help reduce the negative effects of maternal obesity on mothers and offspring (7, 8). Work from our laboratory and others has shown benefits of voluntary exercise before and during pregnancy on maternal and offspring metabolic outcomes associated with diet-induced obesity in rodents (9–11), but the underlying molecular mechanisms are not clear.

Over the last few decades, gut microbiota dysbiosis (perturbations in gut microbiota composition) has been implicated as an important factor contributing to various diseases including obesity, inflammatory bowel diseases, non-alcoholic fatty liver disease and gastrointestinal malignancies (12). Diet is intimately linked to obesity, and known to be a major factor influencing the human gut microbial composition (13). The infant gut is colonized by maternal gut microbes during gestation *in-utero*, and then further through delivery and lactation (14–17). Thus, it is likely that maternal gut microbiota dysbiosis during pregnancy and lactation constitutes a mechanism by which the effects of maternal obesity or over nutrition are conferred to the offspring. In humans, children born to women who were overweight or obese during pregnancy vs. those from normal-weight mothers showed significant differences in gut microbiota composition at 1 month, 6 months (18) and 2 years of age (19). Moreover, gut bacterial populations previously associated with obesity such as *Bacteroides, Oscillibacter*, and *Blautia* species were altered in newborns of mothers who reported a higher intake of fat during pregnancy (20). Similarly, across different animal models, consumption of a high-fat/western-style diet (HFD) during pregnancy and lactation has been associated with offspring gut microbiota dysbiosis (21–24). However, very few studies have analyzed gut microbial communities in both maternal and offspring specimens, essential to establish a direct link between microbial profiles.

Virtually no studies have investigated the impact of maternal diet and exercise prior to and during pregnancy on the gut microbiome of both mothers and their offspring. Recent studies in male rodents have explored the impacts of HFD and exercise on the gut microbiome. For instance, voluntary exercise by male mice could induce shifts in major bacterial phyla (Bacteroidetes and Firmicutes) and prevent weight gain and adiposity associated with HFD feeding (25). Voluntary or forced exercise altered the gut microbiota composition and these were orthogonal to alterations induced by HFD feeding in male mice (26, 27). But these studies were limited to the use of sequencing techniques such as denaturing gradient gel electrophoresis which only detect predominant bacterial members in the gut microbiome. In this study we sought to determine whether prolonged feeding of obesogenic diet and voluntary exercise prior to and during pregnancy were associated with changes in the gut microbiomes of Sprague-Dawley rat dams and their offspring. To investigate this, 16S rRNA gene sequencing was employed on feces collected from dams and their male and female offspring around the time of weaning, alongside detailed anthropometric and blood and tissue sampling for metabolic measures. We also investigated whether changes in the maternal and offspring gut microbiota composition were associated with metabolic measures at the time of sampling.

## Results

### Obesogenic diet had impacts on maternal gut microbiota α-diversity and composition

**Figure 1:**
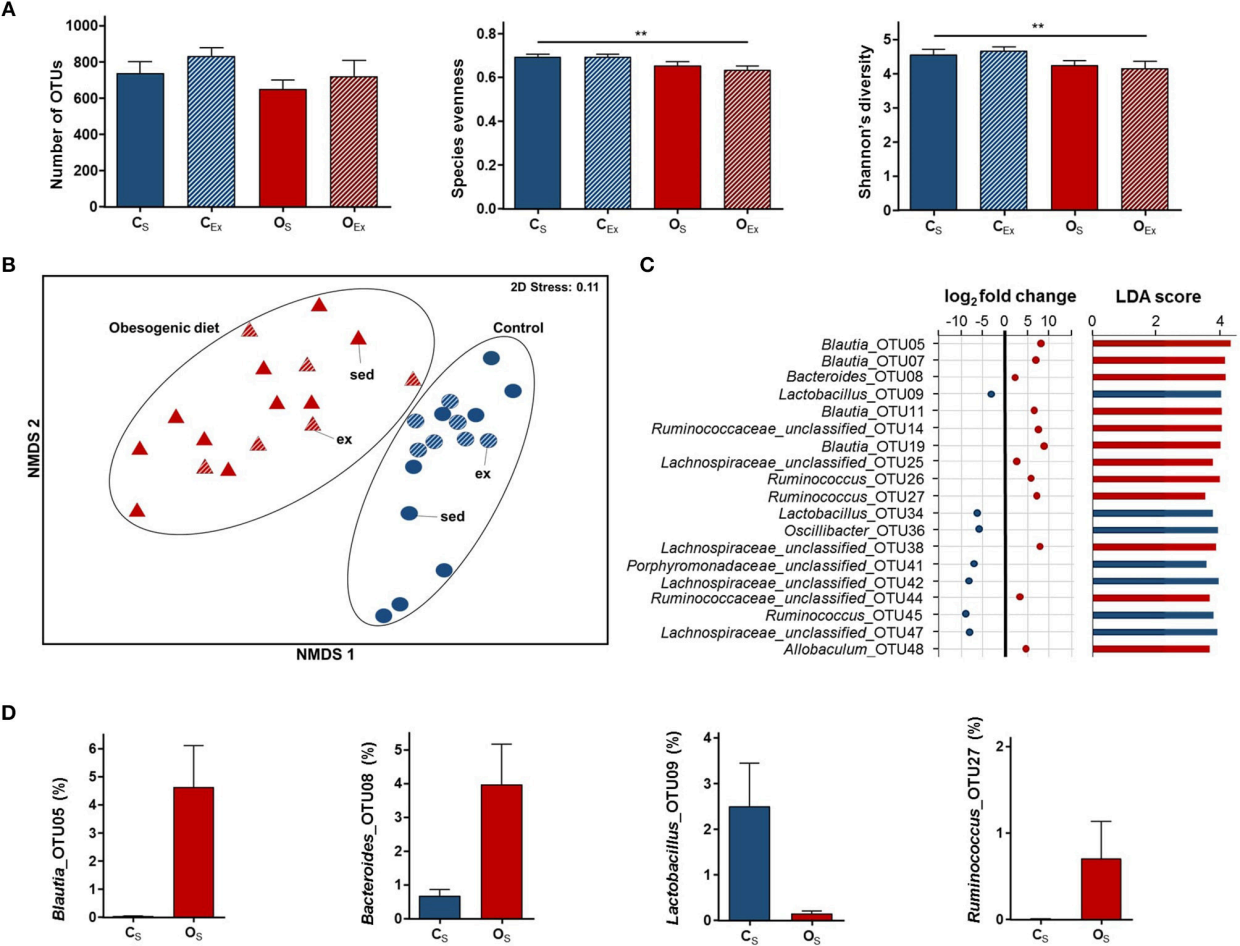
Impact of obesogenic diet on maternal gut microbiome. **(A)** Measures of α-diversity (number of operational taxonomic units (OTUs), species evenness, and Shannon's diversity index). Data are displayed as mean ± SEM and were analyzed by two-way ANOVA. Main effects are indicated on top of horizontal line; ^**^p ≤ 0.01 maternal diet effect. **(B)** Non-metric multidimensional scaling (NMDS) plot following square root transformation and Bray-Curtis resemblance of relative abundance data at the OTU level. Maternal exercise is denoted as sed (sedentary) and ex (exercised). **(C)** Microbial taxa among top 50 OTUs identified to be significantly different in abundance between O_S_ and C_S_ dams by DESeq2 (*p* ≤ 0.05) and LEfSe (LDA Score>2.0, *p* ≤ 0.05) analyses. In DESeq2, negative log_2_ fold change value denotes decreased abundance and positive log_2_ fold change value denotes increased abundance in O_S_ dams relative to C_S_ dams. **(D)** Relative abundance of four microbial taxa identified to be differentially abundant between O_S_ and C_S_ dams by DESeq2 and LEfSe analyses. Data are displayed as mean ± SEM. C_S_ (control diet sedentary; blue): *n* = 9, C_Ex_ (control diet exercised; striped blue): *n* = 7, O_S_ (obesogenic diet sedentary; red): *n* = 10, and O_Ex_ (obesogenic diet exercised; striped red): *n* = 6.

We previously reported that at 4 weeks post-partum, dams consuming obesogenic diet had higher plasma triglyceride and insulin concentrations, increased body weight and adiposity relative to those on control diet (10). There was no significant difference in blood glucose concentrations between diet groups (10). To analyse impacts of diet and exercise on the maternal gut microbiota, we performed 16S rRNA amplicon sequencing on fecal samples collected from dams at this time (control diet sedentary: C_S_, control diet exercised: C_Ex_, obesogenic diet sedentary: O_S_, and obesogenic diet exercised: O_Ex_). We first examined several α-diversity measures across maternal diet groups. Both species evenness [*F*_(1, 28)_ = 11.1, *p* = 0.002; C_S_: 0.69 ± 0.01, C_Ex_: 0.69 ± 0.01, O_S_: 0.65 ± 0.02, and O_Ex_: 0.63 ± 0.02] and Shannon's diversity index [*F*_(1, 28)_ = 8.1, *p* = 0.008; C_S_: 4.60 ± 0.13, C_Ex_: 4.70 ± 0.10, O_S_: 4.20 ± 0.14, and O_Ex_: 4.20 ± 0.20] were significantly lower in dams fed obesogenic diet relative to control dams (Figure 1A). There was no significant difference in the number of OTUs between diet groups [*F*_(1, 28)_ = 2.6, *p* = 0.12; C_S_: 738.4 ± 63.5, C_Ex_: 829.3 ± 47.2, O_S_: 649.2 ± 47.6, and O_Ex_: 718.0 ± 88.1; Figure 1A).

To determine whether the overall gut microbial composition of obesogenic and control diet fed dams were different, we examined different β-diversity measures. Dams fed obesogenic diet clustered differently to control dams at the OTU level as observed by NMDS (Figure 1B). PERMANOVA analyses likewise confirmed significant differences in β-diversity between diet groups (*t* = 2.6, *df* = 1, 30, *p* = 0.001). Maternal diet did not alter sample dispersions (variation of Bray-Curtis similarities; PERMDISP: *t* = 1.0, *df* = 1, 30, *p* = 0.35).

We next examined sedentary dams to identify individual microbial taxa that differed between diet groups at the OTU level. LEfSe analysis identified 292 differentially abundant OTUs, while DESeq2 analysis identified 167 differentially abundant OTUs between diet groups. A total of 131 differentially abundant OTUs was consistent across both analyses, with 60 OTUs enriched and 71 OTUs decreased in O_S_ dams when compared to C_S_ dams. Several OTUs affiliated with genera *Lactobacillus, Alistipes*, and unclassified genera within *Porphyromonadaceae* and *Lachnospiraceae* families were decreased in O_S_ dams. On the other hand, OTUs affiliated with *Blautia, Ruminococcus* and *Bacteroides* were more abundant in O_S_ dams (Figures 1C,D). LEfSe analysis at the higher taxonomic levels identified *Lactobacillus, Blautia* and *Bacteroides* genera as differentially abundant between O_S_ and C_S_ dams (LDA score>4), which was consistent with the OTU results.

### Maternal gut microbiota composition was associated with metabolic measures

Distance based linear models (DistLM) were used to identify associations between the overall maternal gut microbial composition and several metabolic parameters. The metabolic parameters tested were: fat mass, blood glucose concentrations, plasma leptin, insulin and triglyceride (TG) concentrations at endpoint, and change in body weight over the experiment. The analysis was performed between metabolic parameters and a Bray-Curtis resemblance matrix of the overall gut microbiome at the OTU level. All the considered parameters, except for blood glucose, were significantly associated with the overall gut microbial composition at the OTU level (Table 1).

**Table 1:** Correlations between overall maternal gut microbial composition and metabolic parameters.

**Variable**	**Pseudo-*F***	**p(perm)**	**res.df**
Leptin	3.3	**0.001**	30
Insulin	1.8	**0.015**	30
Glucose	0.69	0.94	30
Triglycerides	4.3	**0.001**	30
Fat mass	4.6	**0.001**	30
Change in body weight	3.2	**0.002**	30
Distance run	1.4	0.11	30

*Distance based linear modeling (DistLM) was performed using a Bray-Curtis resemblance matrix of the overall gut microbiome composition at the operational taxonomic unit level with metabolic parameters: plasma leptin, insulin, triglyceride and blood glucose concentrations, fat mass, and change in body weight over the experiment as predictor variables. Fat mass was measured as the sum of retroperitoneal, visceral and inguinal fat pads. Distance run represented average kilometers run per day by each rat. A forward-stepping selection procedure and Akaike Information Criterion selection criterion was used. Pseudo-F and p-values were obtained using 999 permutations (perm) and bold values indicate significance, p ≤ 0.05*.

*res.df = residual degrees of freedom*.

Further, we performed DistLM analyses to investigate whether specific microbial taxa were associated with these metabolic parameters. Here the analysis was performed between a Euclidean distance resemblance matrix of each parameter and the relative abundances of the top 150 OTUs. Several OTUs differentially abundant with diet were associated with the tested parameters. *Lactobacillus*_OTU34 (decreased 6-fold in O_S_ dams), *Blautia_*OTU5 (enriched 8-fold in O_S_ dams) and *Bacteroides*_OTU8 (enriched 3-fold in O_S_ dams) were associated with all tested metabolic parameters except blood glucose and insulin. *Ruminococcus*_OTU27 (enriched more than 7-fold in O_S_ dams) was associated with insulin and leptin. Only *Lachnospiraceae unclassified_*OTU47 (decreased 8-fold in O_S_ dams) was associated with blood glucose.

### Maternal exercise altered gut microbial composition in control dams

Dams had access to running wheels from 10 days prior to mating until delivery, and exercise levels did not differ between diet groups (10). At the time of sampling, maternal exercise had no discernible metabolic impacts in obesogenic or control diet dams at 4 weeks post-partum (10), however, it is possible that some impacts were present in the gut microbiota. We identified no significant effect of maternal exercise on α-diversity measures (species evenness, number of OTUs, or Shannon's diversity index; Figure 1A) in the dams.

To determine whether maternal exercise had an impact on the overall gut microbial composition, PERMANOVA analysis was conducted between exercised and sedentary dams in each diet group, separately (C_Ex_ vs. C_S_, O_Ex_ vs. O_S_). Pairwise PERMANOVA revealed C_Ex_ dams had significantly different gut microbial compositions compared to C_S_ dams (*t* = 1.2, *df* = 14, *p* = 0.041), in contrast, there was no significant difference between O_Ex_ and O_S_ dams (*t* = 0.98, *df* = 14, *p* = 0.50). The microbial communities of sedentary dams were more dispersed than those of exercised dams (PERMDISP: *t* = 3.2, *df* = 1, 30, *p* = 0.009). We also tested whether average distance run per day was associated with the gut microbial composition, and found no significant correlation (Table 1).

We then applied LEfSe and DESeq2 analyses between sedentary and exercised dams in each diet group, separately. Consistent across both analyses, one differentially abundant OTU was identified between C_Ex_ and C_S_ dams, while no differentially abundant OTUs were identified between O_Ex_ and O_S_ dams. *Anaerostipes_*OTU70 was decreased in C_Ex_ dams relative to C_S_ dams (DESeq2: log_2_ fold change = −8.6, *p* = 0.0018; LEfSe: LDA>3, *p* = 0.018). At the higher taxonomic levels, LEfSe identified genera that were not affiliated with OTUs differentially abundant with maternal exercise such as *Clostridium*_XlVa (decreased in C_Ex_ relative to C_S_; LDA>4) and *Clostridium*_XVIII (decreased in O_Ex_ relative to O_S_; LDA>3).

### Offspring gut microbiota α-diversity measures were impacted by maternal diet and exercise

**Figure 2:**
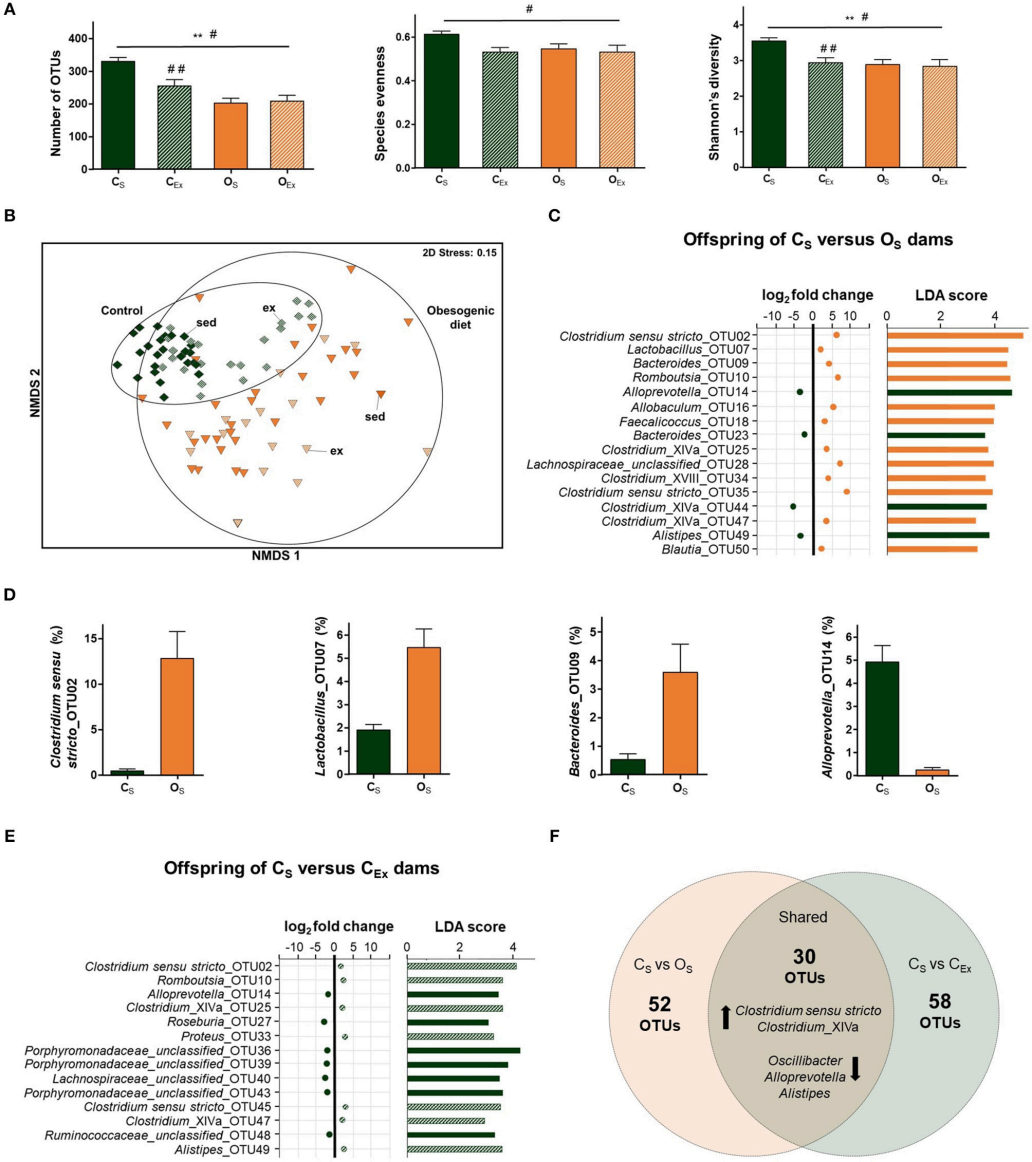
Impacts of maternal obesogenic diet and voluntary exercise on offspring gut microbiome. **(A)** Measures of α-diversity (number of operational taxonomic units (OTUs), species evenness, and Shannon's diversity index). Data are displayed as mean±SEM and were analyzed by two-way ANOVA. Main effects indicated on top of horizontal line; **p ≤ 0.01 maternal diet effect; ^#^*p* ≤ 0.05 maternal exercise effect. Simple main effects are indicated on top of bar; ^##^*p* ≤ 0.01 maternal exercise effect. **(B)** Non-metric multidimensional scaling (NMDS) plot following square root transformation and Bray-Curtis resemblance of relative abundance at the OTU level. Maternal exercise is denoted as sed (sedentary) and ex (exercised). **(C)** Microbial taxa among top 50 OTUs identified to be significantly different in abundance between offspring of O_S_ and C_S_ dams by DESeq2 (*p* ≤ 0.05) and LEfSe (LDA Score>2.0, *p* ≤ 0.05) analyses. In DESeq2, negative log_2_ fold change value denotes decreased abundance and positive log_2_ fold change value denotes increased abundance in offspring of O_S_ relative to C_S_. **(D)** Relative abundance of four microbial taxa identified to be differentially abundant between offspring of O_S_ and C_S_ dams by DESeq2 and LEfSe analyses. Data are displayed as mean ± SEM. **(E)** Microbial taxa among top 50 OTUs identified to be significantly different in abundance between offspring of C_Ex_ and C_S_ dams by DESeq2 (*p* ≤ 0.05) and LEfSe (LDA Score>2.0, *p* ≤ 0.05) analyses. In DESeq2, negative log_2_ fold change value denotes decreased abundance and positive log_2_ fold change value denotes increased abundance in offspring of C_Ex_ relative to C_S_. **(F)** Number of OTUs altered in a similar direction in offspring of both C_Ex_ (green) and O_S_ (orange) dams relative to offspring of C_S_ dams. Black arrows denote either increase or decrease in abundance of microbial taxa. Offspring from C_S_ (control diet sedentary; green): *n* = 25, C_Ex_ (control diet exercised; striped green): *n* = 22, O_S_ (obesogenic diet sedentary; orange): *n* = 29, and O_Ex_ (obesogenic diet exercised; striped orange): *n* = 17.

The gut microbiota of male and female offspring at postnatal day (PND) 19 was examined using 16S rRNA amplicon sequencing. Maternal obesogenic diet was associated with higher blood glucose concentrations, final body weight and adiposity in offspring at PND19 (10). Maternal diet and exercise had sex-specific effects on blood glucose concentrations, plasma insulin and TG concentrations in the offspring (10). In male offspring, maternal exercise was associated with significantly lower blood glucose concentrations (O_Ex_ vs. O_S_ offspring). Insulin concentrations were lower in male offspring of both C_Ex_ and O_Ex_ dams, with no significant effect of maternal diet. In contrast, insulin concentrations were higher in female offspring of O_S_ vs. C_S_ dams, with no significant effect of maternal exercise. Plasma TG concentrations were significantly lower in male offspring of C_Ex_ vs. C_S_ dams. There were no significant effects of maternal exercise on blood glucose concentrations, plasma insulin or TG concentrations in female offspring of obesogenic and control diet dams (10). Here, we found no sex-specific differences in α-diversity measures (three-way ANOVA: offspring sex, maternal diet and maternal exercise). Thus, data from both sexes was aggregated to examine differences in α-diversity across diet and exercise. A two-way ANOVA indicated a significant interaction between maternal diet and exercise on the number of OTUs [*F*_(1, 89)_ = 7.4, *p* = 0.008; C_S_: 330.5 ± 10.3, C_Ex_: 255.0 ± 19.3, O_S_: 203.0 ± 13.5, and O_Ex_: 209.5 ± 15.8] and Shannon's diversity index [*F*_(1, 89)_ = 4.6, *p* = 0.034; C_S_: 3.60 ± 0.08, C_Ex_: 2.90 ± 0.13, O_S_: 2.90 ± 0.13, and O_Ex_: 2.80 ± 0.18] in offspring. Simple main effects analysis indicated that maternal exercise only significantly decreased both α-diversity measures in offspring of control dams (*p* ≤ 0.001 for both), as shown in Figure 2A. Maternal exercise, across diet groups, significantly decreased species evenness [*F*_(1, 89)_ = 5.8, *p* = 0.018; C_S_: 0.61 ± 0.01, C_Ex_: 0.53 ± 0.02, O_S_: 0.55 ± 0.02, and O_Ex_: 0.53 ± 0.03], with no significant effect of maternal diet (Figure 2A).

### Maternal obesogenic diet altered offspring gut microbiota composition

No sex-specific differences in gut microbial composition were identified globally (PERMANOVA: *t* = 0.78, *df* = 1, 91, *p* = 0.66), within each sub-group (PERMANOVA: C_S_: *t* = 0.74, *df* = 1, 23, *p* = 0.72, C_Ex_: *t* = 0.99, *df* = 1, 20, *p* = 0.39, O_S_: *t* = 0.84, *df* = 1, 27, *p* = 0.54, and O_Ex_: *t* = 0.62, *df* = 1, 15, *p* = 0.80), or in abundance of individual microbial taxa within each sub-group (LEfSe and DESeq2 analyses) between male and female offspring. Thus, data from both sexes was aggregated to examine differences in β-diversity across diet and exercise. The microbial compositions of offspring from obesogenic diet dams differed significantly when compared to offspring from control dams, as visualized by NMDS (Figure 2B), and confirmed using PERMANOVA analyses (*t* = 3.5, *df* = 1, 91, *p* = 0.001). Microbial communities in offspring from control dams were less dispersed than in offspring from obesogenic diet dams (PERMDISP: *t* = 4.5, *df* = 1, 91, *p* = 0.001).

LEfSe and DESeq2 analyses were employed on microbial abundance data from offspring of sedentary dams across both diet groups. Of the 394 differentially abundant OTUs detected by LEfSe, 82 were consistent with DESeq2 analysis. More OTUs were decreased (53 OTUs) than enriched (29 OTUs) in O_S_ relative to C_S_ offspring. OTUs affiliated with *Clostridium sensu stricto, Clostridium_*XlVa, *Bacteroides, Blautia* and *Lactobacillus* were enriched, while *Alistipes, Oscillibacter* and *Alloprevotella* OTUs were decreased in O_S_ offspring (Figures 2C,D). Consistent with the OTU results, LEfSe found *Clostridium sensu stricto* and *Lactobacillus* genera were enriched, while *Alloprevotella* was decreased in O_S_ vs. C_S_ offspring (LDA score>4).

### Maternal exercise altered offspring gut microbiota composition in a maternal diet dependent manner

The impact of maternal exercise on β-diversity was calculated between offspring of exercised and sedentary dams in each diet group, separately. PERMANOVA analyses revealed significantly different gut microbial compositions in offspring from C_Ex_ vs. C_S_ dams (*t* = 2.4, *df* = 45, *p* = 0.001). There was no significant difference in β-diversity between offspring from O_Ex_ and O_S_ dams (*t* = 1.1, *df* = 44, *p* = 0.20). The dispersion of microbial communities was similar between offspring from exercised and sedentary dams (PERMDISP: *t* = 0.46, *df* = 1, 91, *p* = 0.67).

We then investigated whether maternal exercise altered the abundance of specific microbial taxa in the offspring. LEfSe and DESeq2 identified 88 differentially abundant OTUs between C_Ex_ and C_S_ offspring. In contrast, no differentially abundant OTUs were identified between offspring of O_Ex_ and O_S_ dams. OTUs affiliated with genera *Romboutsia, Clostridium sensu stricto* and *Proteus* were enriched, whereas OTUs affiliated with *Roseburia, Alistipes* and *unclassified genera within Porphyromonadaceae* and *Lachnospiraceae families* were decreased in C_Ex_ offspring (Figure 2E). Of the 88 differentially abundant OTUs between C_Ex_ and C_S_ offspring, 30 were also differentially abundant between O_S_ vs. C_S_ offspring, as shown in Figure 2F. In both O_S_ and C_Ex_ offspring, OTUs affiliated with *Clostridium sensu stricto* and *Clostridium_*XlVa were enriched, while OTUs affiliated with *Alloprevotella, Alistipes* and *Oscillibacter* were decreased relative to C_S_ offspring (Figure 2F).

### Offspring gut microbiota composition was associated with metabolic measures

DistLM was used to analyse the association between the overall gut microbial composition and metabolic parameters in the offspring. The analysis was performed against metabolic parameters: final body weight, visceral fat, and blood glucose concentrations, as well as plasma leptin, and insulin and TG concentrations. The overall offspring gut microbial composition (Bray-Curtis resemblance matrix) was significantly associated with final body weight, visceral fat, glucose and leptin (Table 2).

**Table 2:** Correlations between overall offspring gut microbial composition and metabolic parameters.

**Variable**	**Pseudo-*F***	***p*(perm)**	**Res.df**
Leptin	4.1	**0.001**	87
Insulin	1.6	0.069	85
Glucose	2.9	**0.005**	91
Triglycerides	1.5	0.086	79
Visceral fat	10.6	**0.001**	91
Final body weight	10.6	**0.001**	91

*Distance based linear modeling (DistLM) was performed using a Bray-Curtis resemblance matrix of the overall gut microbiome composition at the operational taxonomic unit level with metabolic parameters: plasma leptin, insulin, triglyceride and blood glucose concentrations, visceral fat, and final body weight as predictor variables. A forward-stepping selection procedure and Akaike Information Criterion selection criterion was used. Pseudo-F and p-values were obtained using 999 permutations (perm) and bold values indicate significance, p ≤ 0.05*.

*res.df = residual degrees of freedom*.

The top 150 OTUs whose relative abundance was differentially affected by maternal diet and exercise were analyzed for associations with final body weight, visceral fat and blood glucose (Euclidean distance resemblance matrix) using DistLM. OTUs enriched in O_S_ offspring such as *Lactobacillus_*OTU7, *Blautia*_OTU54 and *Bacteroides*_OTU9 were associated with all the tested metabolic parameters. We also identified associations for OTUs altered in both O_S_ offspring (O_S_ vs. C_S_) and C_Ex_ offspring (C_Ex_ vs. C_S_) with metabolic parameters. *Clostridium sensu stricto*_OTU2 and *Clostridium* XlVa*_*OTU25 (enriched in O_S_ and C_Ex_ offspring) were associated with final body weight. *Alloprevotella*_OTU14, *Alistipes*_OTU49, and *Oscillibacter*_OTU55 (decreased in O_S_ and C_Ex_ offspring) were associated with all the tested metabolic parameters.

### Predicted functions of offspring gut microbiota were altered by maternal diet and exercise

In order to infer changes in microbial metabolic pathways in the offspring gut microbiota resulting from maternal diet or exercise, functional content was predicted from amplicon data using PICRUSt. All offspring samples had low nearest sequenced taxon index (NSTI) values (average NSTI: 0.058 ± 0.003) indicating high prediction accuracy. Several predicted pathways were significantly enriched in offspring from obesogenic vs. control diet dams (Bonferroni corrected ANOVA with maternal exercise status nested as subclass, *p* < 0.05), among them, fructose and mannose metabolism (Table 3). In contrast, pathways related to indole alkaloid biosynthesis, α-linolenic acid metabolism and carotenoid metabolism were significantly decreased (Table 3). No significantly different pathways were identified after FDR or Bonferroni correction for maternal exercise (ANOVA with maternal diet type nested as subclass). We then analyzed offspring from control diet dams separately (offspring of C_Ex_ vs. C_S_ dams). Pathways related to vasopressin regulated water reabsorption (fold change = −3.0, *p* = 0.00064, *q* = 0.067), indole alkaloid biosynthesis (fold change = −2.2, *p* = 0.0011, *q* = 0.067), and betalain biosynthesis (fold change = −2.2, *p* = 0.0011, *q* = 0.067) were decreased, while those related to phosphotransferase system (fold change = 1.5, *p* = 0.00083, *q* = 0.067), and *Staphylococcus aureus* infection (fold change = 1.9, *p* = 0.0013, *q* = 0.067) were increased in C_Ex_ offspring.

**Table 3:** Predicted bacterial pathways altered by maternal diet in the offspring gut microbiota.

**Pathway**	**Fold-change**	**Mean (C)**	**Mean (O)**	***P* (ANOVA)**	***P*(adj)**
Alpha Linolenic acid metabolism	−2.016	4203.68	2084.87	0.00000014	0.000038
Amyotrophic lateral sclerosis	−1.683	7158.94	4253.39	0.00000021	0.000057
Carotenoid biosynthesis	−2.165	9428	4354.37	0.00000091	0.00025
Vasopressin regulated water reabsorption	−5.581	8.98	1.61	0.0000014	0.00038
Geraniol degradation	−1.724	20611.09	11953.09	0.0000023	0.00063
Caprolactam degradation	−1.88	18865.79	10035.26	0.0000024	0.00065
Lipopolysaccharide biosynthesis	−1.654	77024.87	46576.93	0.0000026	0.00071
Lipopolysaccharide biosynthesis proteins	−1.547	119476.9	77230.09	0.0000033	0.0009
Shigellosis	−2.196	3437.06	1564.85	0.0000045	0.0012
Indole alkaloid biosynthesis	−16.09	6.3	0.39	0.0000046	0.0013
Bladder cancer	−2.172	1734.15	798.35	0.0000054	0.0015
Pertussis	−1.702	10961.15	6439.37	0.0000055	0.0015
Prion diseases	−1.653	2211.87	1338.09	0.0000068	0.0018
Bacterial invasion of epithelial cells	−2.125	5215.64	2454.93	0.0000073	0.002
Betalain biosynthesis	−11.14	6.3	0.57	0.0000098	0.0027
Metabolism of xenobiotics (cytochrome P450)	−1.705	12248.57	7182.28	0.00001	0.0027
Chlorocyclohexane and chlorobenzene degradation	−1.707	9778.04	5726.59	0.000016	0.0044
Drug metabolism (cytochrome P450)	−1.697	13039.94	7683.5	0.000019	0.0052
Sporulation	1.71	90806.6	155265.2	0.000044	0.012
Fructose and mannose metabolism	1.362	214300.3	291772.5	0.00015	0.041
Inositol phosphate metabolism	−1.377	35218.09	25582.15	0.00017	0.046
Restriction enzyme	1.343	26422.96	35483.37	0.00018	0.049

*Nested ANOVA was performed using KEGG level 3 pathway counts generated by PICRUSt. The main variable was diet with exercise nested within. P-values were adjusted (Padj) with Bonferroni correction and only pathways showing P(adjusted) < 0.05 are shown. Negative fold change value denotes decreased abundance and positive fold change value denotes increased abundance in O_S_ dams relative to C_S_ dams*.

## Discussion

Alterations in the maternal gut microbiota during critical periods of embryonic, fetal, and early postnatal development may have effects on the offspring gut microbiota, with lifelong consequences for susceptibility to disease (28). Our results indicate that chronic consumption of an obesogenic diet prior to and during gestation, and continuing during lactation, induced changes in the maternal and offspring gut microbiota. This affected the predicted profile of microbial metabolic pathways related to lipid and carbohydrate metabolism in the offspring. We identified no marked effects of maternal exercise on the gut microbiota of dams fed obesogenic diet or their offspring. In contrast, maternal exercise decreased α-diversity and altered the abundance of 88 microbial taxa in offspring of C_Ex_ vs. C_S_ dams. Thirty of these taxa were altered in a similar direction in offspring of O_S_ vs. C_S_ dams and associated with several metabolic markers relevant to obesity. We postulate that this dysbiosis may be due to the impacts of catch-up growth in offspring of C_Ex_ dams.

Results of this study emphasize the importance of healthy dietary habits during pregnancy and lactation on maternal and offspring outcomes. We found that consumption of an obesogenic diet induced changes in the maternal gut microbiota and these were transferred to their offspring of both sexes. Our results are in accordance with a metagenomic study in primates that showed HFD feeding during pregnancy and lactation induced changes in the gut microbial composition of mothers and their offspring (22). OTUs affiliated with *Bacteroides* and *Blautia* were enriched in both dams fed obesogenic diet and their offspring. The relative abundances of these OTUs were associated with several measures of adiposity in both dams and offspring. In support of our results, *Bacteroides* was enriched in the gut microbiota of both overweight mothers and their infants at one and 6 months of age compared to infants from normal weight mothers (18). Other studies in humans have associated increased *Bacteroides* with overweight and obesity (29–31). However, decreased *Bacteroides* was reported in newborns of mothers reporting a greater intake of fat during pregnancy (20). Increased levels of *Bacteroides* have also been associated with weight loss in obese human subjects undergoing dietary or surgical interventions (32–34). Variations across these studies can be attributed to differences in sequencing methods, taxonomic level analyzed and diet. Our findings along with previous evidence suggest a role for *Bacteroides* in the modulation of host adiposity, however, a causal link is yet to be established.

We inferred the predicted metabolic functions of the offspring gut microbiota using PICRUSt. Our analyses revealed a clear difference in the predicted functional capability between the microbiota of offspring from obesogenic vs. control diet dams. Specifically, pathways related to fatty acid metabolism such α-linolenic acid metabolism were decreased in offspring from obesogenic diet dams. The metabolism of α-linolenic acid by gastrointestinal microbes is known to generate conjugated fatty acids, which have shown potential to reduce atherosclerosis and adiposity (35). Conversely, pathways involved in carbohydrate metabolism (specifically fructose and mannose metabolism) were more abundant in offspring from obesogenic diet dams. This has also been reported in juvenile primates exposed to maternal HFD during gestation and lactation (22). Further, an increased abundance of pathways related to carbohydrate metabolism is a consistent finding across previous studies from our laboratory (36, 37) and others (38, 39) in adult rodents fed HFD. However, these findings need to be confirmed experimentally.

We previously reported that maternal exercise prior to and during pregnancy limited the detrimental impacts of maternal obesity in offspring by reducing plasma insulin and glucose concentrations at PND19 (10). In the present study, maternal exercise had no significant impact on measures of α-diversity and microbial composition in dams fed obesogenic diet or their offspring. This may be due to the major impact of obesogenic diet on the gut microbiota and the moderate level of exercise achieved by the dams. More marked effects of exercise have been reported in previous studies comparing the effect of HFD and exercise on the gut microbiota in adult male rodents (25–40). This discrepancy may be related to differences in diet and exercise paradigms, and developmental stage that exercise was introduced, as here exercise was confined to pre-pregnancy and during gestation. Our results suggest that maternal exercise prior to and during pregnancy cannot rescue the detrimental effects of a maternal obesogenic diet on the maternal and offspring gut microbiota. The mechanism(s) by which maternal exercise induces metabolic benefits in obesogenic diet fed dams and their offspring warrants further investigation.

In contrast, voluntary maternal exercise via wheel running had significant effects on gut microbiota composition in control dams and their offspring. Though a different exercise regimen was used, our results are supported by a study which reported significant impacts of exercise on gut microbial composition in control male Wistar rats, while there was no impact in those fed HFD (41). Interestingly, we found that of the 88 OTUs altered by maternal exercise in offspring of control dams, 30 were altered in a similar direction in offspring of O_S_ vs. C_S_ dams. Of interest, *Oscillibacter* OTUs were decreased in offspring of both C_Ex_ and O_S_ dams, and these OTUs were associated with blood glucose levels and several measures of adiposity. Other studies have also associated changes in the abundance of *Oscillibacter* with obesity and metabolic parameters. For instance, *Oscillibacter* was negatively correlated with BMI or postprandial glucose area under the curve in humans (42, 43). In monozygotic twins discordant for obesity in terms of BMI, members of *Oscillibacter* were more abundant in twins with lower BMI (44). However, very little is known about the physiological role of this bacterium. Our analyses also revealed similar alterations in microbial metabolic pathways in offspring of both obesogenic diet and control exercised dams. Notably, pathways related to indole alkaloid biosynthesis were decreased in both. The intestinal microbiota has been reported to metabolize tryptophan using tryptophanase to produce indole metabolites (45). It was recently reported that *Lactobacillus murinus* supplementation blunted high salt diet induced T_H_17 activation and ameliorated salt sensitive hypertension in mice, and this was associated with an increase in fecal indole metabolites (46). In weaning piglets, tryptophan supplementation enhanced indole alkaloid biosynthesis pathways and downregulated the expression of inflammatory cytokines and improved intestinal mucosal barrier function (47).

We postulate that gut dysbiosis in offspring of C_Ex_ dams could be a reflection of catch-up growth (accelerated postnatal growth). Maternal exercise by control dams was associated with lower birth weight in their offspring across both sexes and this effect was diminished at weaning (10). Despite evidence in human studies that catch-up growth can increase the risk of metabolic diseases in adulthood (48, 49), very few studies have investigated its effects on the gut microbiome. A recent study in mice identified changes in the gut microbial composition of pups experiencing catch-up growth, and this was associated with excessive adiposity and glucose intolerance in adulthood. Specifically, they found that members of the *Lachnospiraceae* family were decreased in pups experiencing catch-up growth at 4 weeks of age (50), which is similar to our observations in offspring of C_Ex_ dams. Future studies should investigate whether these changes in gut microbial composition in offspring persist into adulthood and increase their susceptibility to metabolic disease.

There are several limitations to our study worth noting. We ceased voluntary exercise after gestation in order to avoid any interruption to nursing behavior by dams during lactation. It is possible some impacts of exercise on the maternal and offspring gut microbiome abated over time or were overridden by diet. In addition, this study did not account for other maternal factors such as the vaginal, breast milk, and skin microbiome, which are also known to impact the offspring gut microbial composition (15, 51). Despite the fact that the analysis between dam and offspring was highly consistent, our use of different primer sets for 16S rRNA gene amplification in dams and offspring may have influenced the results. Studies have detected different changes in gut microbial composition data depending on the region of 16S rRNA gene sequenced (52, 53). Moreover, it is not clear whether our findings can be extended to humans, rodent pups have a very immature intestinal tract at birth and during lactation (day 0–21), whereas in human infants the intestinal tract is more mature at birth (54). Nevertheless, our data in rats is an essential starting point in elucidating the impact of maternal obesogenic diet and voluntary exercise on the gut microbiome, especially since it is difficult to undertake similar studies in humans in a controlled manner.

## Conclusions

This study makes novel contributions to our understanding of diet and exercise as maternal factors influencing the maternal and offspring gut microbiome. Importantly, we showed that bacterial taxa previously associated with obesity were altered by obesogenic diet in dams and their offspring. Maternal exercise prior to and during pregnancy had major impacts on the gut microbiota of offspring from control dams, with reduced gut microbial diversity and alterations in microbial composition. Intriguingly, we found that 30 of the 88 microbial taxa altered in offspring of exercised control dams were also altered in a similar direction in offspring of sedentary obesogenic vs. control diet dams. These findings were likely driven by the effects of catch-up growth in offspring of exercised control dams, but further controlled animal studies are needed to interrogate this potential mechanism.

## Materials and methods

### Maternal obesity and exercise model

This study is based on a cohort of female Sprague-Dawley rats and their offspring previously generated in our laboratory (10). Briefly, 6-week old female rats were assigned either standard laboratory chow (11 kJ/g, 13% fat, 22% protein, and 65% carbohydrate by energy; Gordon's Stockfeeds, NSW, Australia) or obesogenic diet *ad libitum*: two types of high-fat pellet (SF03-020: 20 kJ/g, 43% fat, 16% protein, and 41% carbohydrate by energy, and SF03-002: 22.8 kJ/g, 59% fat, 14% protein, and 27% carbohydrate by energy; Specialty feeds, Australia) supplemented with a selection of three different western foods (selected from cakes, potato chips, biscuits, meat pie, pasta with lard, and oats mixed with condensed milk). After 6 weeks, half the rats from each diet group were randomly assigned to voluntary exercise through introduction of a running wheel, while the other half remained sedentary with a locked wheel. Female rats were mated (with males fed standard chow) after 10 days of exercise. After delivery, dams were transferred to cages with no running wheel in order to prevent any interference of exercise with nurturing of pups, and to better model likely post parturition behavior. Dams were maintained on their pre-pregnancy diet throughout pregnancy and lactation.

The average litter size and male to female ratios were not significantly different across the groups. At postnatal day (PND) 1, litters were adjusted to 12 pups per mother (10); pups were allocated to groups so that litters were equally represented. Feces from the distal colon were collected from pups and dams at 3 and 4 weeks postpartum, respectively, at the time of tissue sampling for anthropometric and blood analyses. Fecal samples were immediately flash frozen in liquid nitrogen following collection and stored at -80°C. For the purposes of this study a subset of dams and pups (range 2–4 pups per dam with equal male to female ratios) were used.

### Fecal DNA extraction

DNA was extracted from the feces of dams (C_S_: *n* = 9, C_Ex_: *n* = 7, O_S_: *n* = 10, and O_Ex_: *n* = 7) using the PowerFecal DNA isolation kit (Catalog number: 12830-50, MO BIO Laboratories, Inc., Carlsbad, CA, USA), and offspring of both sexes (male: C_S_: *n* = 12, C_Ex_: *n* = 11, O_S_: *n* = 13, and O_Ex_: *n* = 11, and female: C_S_: *n* = 13, C_Ex_: *n* = 11, O_S_: *n* = 16, and O_Ex_: *n* = 10) using the PowerSoil DNA Isolation Kit (Catalog number: 12888-100, MO BIO Laboratories, Inc., Carlsbad, CA, USA). Extractions were performed according to the manufacturer's instruction. DNA concentration and quality was measured using a DeNovix DS-11 Spectrophotometer (DeNovix, Inc., Delaware, USA).

### 16S rRNA gene sequencing and taxonomic analysis

The composition of gut microbial communities was analyzed by Illumina amplicon sequencing of the 16S rRNA gene (performed by Ramaciotti Center for Genomics, UNSW Sydney) using DNA from dams (2 × 300 base pair MiSeq chemistry, V1-V3 region, 27F-519R primer pair) and offspring (2 × 250 base pair MiSeq chemistry, V4 region, 515F-806R primer pair). Sequence data were processed using Mothur, version 1.39.1 (55), which included removal of ambiguous bases and homopolymers longer than 15 base pairs, alignment with SILVA database, chimera checking with UCHIME, classification against the latest RDP Ribosomal Database training set (version 16_022016), and removal of singletons. Sequences were clustered into operational taxonomic units (OTU) at 97% nucleotide identity to generate an OTU table with the taxonomy and number of sequences per OTU in each sample. Commands were derived using the Mothur MiSeq SOP (56) and modified as required. The generated OTU table, combined with previously published anthropometric and biochemical measurements (10), were used as input in follow-up analyses.

### Statistical analyses

Mother and offspring samples were rarefied down to 24,626 and 12,291 reads, respectively. OTU tables were standardized by dividing feature read counts by total number of reads in each sample. Standardized data were then square root transformed. All statistical analyses explored sex-specific differences in the offspring. One dam was identified as an outlier by Non-metric multidimensional scaling (NMDS). As 88% of the gut microbial composition in this dam was dominated by two OTUs (OTUs 1 and 2) from the genus *Romboutsia*, the dam and her offspring (2 male and 2 female offspring) were excluded from subsequent analyses.

Alpha diversity (number of OTUs, species evenness and Shannon's diversity index) analyses were performed using Calypso (57). Statistical analyses such as two and three-way ANOVA on diversity measures were performed using SPSS software (SPSS statistics version 22, SPSS Inc., an IBM Company). Simple main effects (with Bonferroni adjustment) was performed in the case of a significant interaction between variables on α-diversity. Results are expressed as mean ± SEM and were considered significant if *p* ≤ 0.05. GraphPad Prism 7 was used to plot diversity results. In bar graphs, symbols above horizontal line indicate main effects: ^**^p ≤ 0.01 maternal diet effect and ^#^*p* ≤ 0.05 maternal exercise effect, while symbols above bar indicate simple main effects: ^##^*p* ≤ 0.01 maternal exercise effect.

NMDS plots, Permutational Multivariate Analysis of Variance (PERMANOVA) and Permutational Analysis of Multivariate Dispersions (PERMDISP) were generated using a Bray-Curtis resemblance matrix on PRIMER (Primer-E Ltd., Plymouth, United Kingdom) (58). To identify OTUs differentially abundant between groups we used two different statistical analyses. Linear Discriminant Analysis (LDA) Effect Size (LEfSe) (59) was performed using the Galaxy web application (60) using default settings. The R package Phyloseq (61) was used for the negative binomial Wald test in DESeq2 (62). *P*-values were adjusted for multiple testing using Benjamini Hochberg false discovery rate correction in DESeq2. LEfSe was performed across all taxonomic levels (phylum to OTU level) whereas DESeq2 was performed at the OTU level. Statistical significance was defined as *p* ≤ 0.05 in both statistical methods. Only OTUs identified by both LEfSe and DESeq2, and those with relative abundances consistently represented across more than 50% of rats in a group were discussed.

Distance-based linear models (DistLM) analysis was performed using PRIMER to examine associations between the gut microbiota and metabolic parameters. When the analysis was performed between the overall gut microbial composition and metabolic parameters, a Bray-Curtis resemblance matrix of the overall gut microbial composition at the OTU level was used. The relative abundances of the top 150 OTUs and Euclidean distance resemblance matrix of each metabolic parameter was used to find associations between specific OTUs and metabolic parameters. In both cases DistLM was performed using a forward-stepping selection procedure and Akaike Information Criterion selection criterion (999 permutations). Phylogenetic Investigation of Communities by Reconstruction of Unobserved States (PICRUSt) was performed using Galaxy web to generate a profile of putative functions (through metagenomic prediction) from the 16S rRNA OTU data (63). Taxonomic classification was performed against Greengenes 13.5, and pathway counts were compared across groups using nested ANOVA with Bonferroni correction. Subgroup comparisons were conducted using Wilcoxon test with FDR (FDR corrected *p*-values represented as q).

## Availability of data and material

The sequence data are available in the European Nucleotide Archive (ENA) under accession number PRJEB26886 (http://www.ebi.ac.uk/ena/data/view/PRJEB26886).

## Ethics statement

This study was carried out in accordance with the recommendations of the Animal Research Act 1985 (NSW), the Animal Research Regulations 2010 (NSW), and the NHMRC Australian Code for the Care and Use of Animals for Scientific Purposes 2013 (8th edition). The protocol was approved by the University of New South Wales Animal Care and Ethics Committee (Approval No. 11/104B).

## Author contributions

MM conceived and designed experiments. MM and NK supervised the project. MR, HB, and MM developed the rat cohort. SB, NK, and MM analyzed and interpreted data, and wrote the paper. MR and HB performed all anthropometric and metabolic measurements, and reviewed the paper.

### Conflict of interest statement

The authors declare that the research was conducted in the absence of any commercial or financial relationships that could be construed as a potential conflict of interest.

## Abbreviations

BMI: Body mass index
DNA: Deoxyribonucleic acid
LEfSe: Linear discriminant analysis (LDA) effect size
OTU: Operational taxonomic units
rRNA: Ribosomal RNA gene
PERMANOVA: Permutational multivariate analysis of variance
PND: Postnatal day
O_Ex_: Obesogenic diet exercised
O_S_: Obesogenic diet sedentary
C_Ex_: Control diet exercised
C_S_: Control diet sedentary
NMDS: Non-metric multidimensional scaling
DistLM: Distance-based linear models
SEM: Standard error of mean
PERMDISP: Permutational Analysis of Multivariate Dispersions
HFD: High-fat diet
TG: Triglycerides
perm: Permutations
df: Degrees of freedom
PICRUSt: Phylogenetic Investigation of Communities by Reconstruction of Unobserved States.
